# What Are Lightness Illusions and Why Do We See Them?

**DOI:** 10.1371/journal.pcbi.0030180

**Published:** 2007-09-28

**Authors:** David Corney, R. Beau Lotto

**Affiliations:** UCL Institute of Ophthalmology, University College London, London, United Kingdom; University College London, United Kingdom

## Abstract

Lightness illusions are fundamental to human perception, and yet *why* we see them is still the focus of much research. Here we address the question by modelling not human physiology or perception directly as is typically the case but our natural visual world and the need for robust behaviour. Artificial neural networks were trained to predict the reflectance of surfaces in a synthetic ecology consisting of 3-D “dead-leaves” scenes under non-uniform illumination. The networks learned to solve this task accurately and robustly given only ambiguous sense data. In addition—and as a direct consequence of their experience—the networks also made systematic “errors” in their behaviour commensurate with human illusions, which includes brightness contrast and assimilation—although assimilation (specifically White's illusion) only emerged when the virtual ecology included 3-D, as opposed to 2-D scenes. Subtle variations in these illusions, also found in human perception, were observed, such as the asymmetry of brightness contrast. These data suggest that “illusions” arise in humans because (i) natural stimuli are ambiguous, and (ii) this ambiguity is resolved empirically by encoding the statistical relationship between images and scenes in past visual experience. Since resolving stimulus ambiguity is a challenge faced by all visual systems, a corollary of these findings is that human illusions must be experienced by all visual animals regardless of their particular neural machinery. The data also provide a more formal definition of illusion: the condition in which the true source of a stimulus differs from what is its most likely (and thus perceived) source. As such, illusions are not fundamentally different from non-illusory percepts, all being direct manifestations of the statistical relationship between images and scenes.

## Author Summary

Sometimes the best way to understand how the visual brain works is to understand why it sometimes does not. Thus, visual illusions have been central to the science and philosophy of human consciousness for decades. Here we explain the root cause of brightness illusions, not by modelling human perception or its assumed physiological substrate (as is more typically done), but by modelling the basic challenge that all visual animals must resolve if they are to survive: the inherent ambiguity of sensory data. We do this by training synthetic neural networks to recognise surfaces under different lights in scenes with naturalistic structure. The result is that the networks not only solve this task robustly (i.e., they exhibit “lightness constancy”), they also—as a consequence—exhibit the same illusions of lightness that humans also see. In short, these *synthetic* systems not only get it right like we do, but also get it wrong like we do, too. This emergent coincidence strongly provides causal evidence that illusions (and by extension all percepts) represent the probable source of images in past visual experience, which has fundamental consequences for explaining how and why we see what we do. The study also suggests the first formal definition of what an illusion is: The condition in which the actual source of a stimulus differs from its most likely source.

## Introduction

Understanding how we generate accurate perceptions of surfaces is often best informed by understanding why we sometimes do not. Thus, illusions of lightness (and colour) are essential tools to vision research. In many natural environments, light levels vary across space and over time. It is important to be able to perceive surfaces independently of this varying light intensity (and vice versa) in order to forage or predate successfully, for example. (By reflectance, we mean the proportion of incident light reflected by a surface; lightness is the *perceived* reflectance of a surface; brightness is the *perceived* intensity of light reaching the eye; and luminance is the actual intensity of the light that reaches the eye with respect to the sensitivity of the human visual system.)

A number of models of lightness perception have been proposed, but most of these fail to deal with complex stimuli or only demonstrate a narrow range of behaviours. For instance, one well-known heuristic model predicts human lightness perceptions by first subdividing stimuli into multiple “local frameworks” based on, for instance, junction analysis, and co-planarity as well as other classic gestalt factors. Then, within each framework, the ratio of a patch's intensity and the maximum intensity in that patch's local framework is used to predict the reflectance, combining a “bright is white” and a “large is white” area rule [1]. These rules are well-defined and effective for simple stimuli (e.g., with two nonzero luminance regions), but the application of the rule has not been studied for more complex images [1]. Indeed, it is hard to see how such a model could be applied to even moderately complex stimuli, much less natural scenes under spatially heterogeneous illumination, without extremely complex edge-classification rules that are as yet undefined. Furthermore, such human-based heuristics provide little insight into the physiological and/or computational principles of vision that are relevant to all visual animals.

More computational approaches, on the other hand, are less descriptive, more quantitative, and make fewer assumptions. For example, artificial neural networks (ANNs) have been trained to extract scene information, such as object shape and movement, from simple synthetic images [2,3]; and a statistical approach using Gibbs sampling and Markov random fields has been used to separate reflectance and illumination from simple images [4]. Most such models, however, are unable to explain brightness contrast and assimilation (e.g., White's illusion) simultaneously without recourse to one or more adjustable weighting factors. One approach that can is the Blakeslee and McCourt filter model [5]. By applying a set of filters (specifically, a bank of oriented difference of Gaussians filters, or ODOG), the model produces results that correspond closely to psychophysical results on a wide range of illusory stimuli. The same model, however, fails to predict the asymmetry of brightness contrast, where darker surrounds cause larger illusions than equally lighter surrounds, as we discuss later. “While these asymmetries are not captured by the ODOG model as it is presently implemented, permitting different gain parameters to be applied to the outputs of independent on-channels and off-channels would constitute a logical first step toward accommodating these differences” [6]. It is also important to stress that the model does not attempt to predict the reflectance of surfaces, only the perceived brightness of a stimulus, and therefore is unable to explain lightness constancy in more natural scenes under spatially heterogeneous illumination. Related machine vision work includes the separation of luminance changes into those caused by shading (including the slant of the surface and direction of incident light), and those caused by paint on the surface, using filters and a mixture of Gaussians [7]; and a localised “mixture of experts” and a set of multiscale filters has been used to extract the intrinsic components of an image, including “de-noising” it [8]. However, these studies do not attempt to explain the human perception of lightness or illusions. Thus, explanations as to why and how we see lightness illusions remain incomplete.

Here we take a different approach to rationalising human illusions and, by extension, lightness perception generally. Rather than modelling human perception or known primate physiology—as is typical of most models—we instead model the empirical process by which vision resolves the most fundamental challenge of visual ecology: the inherent ambiguity of visual stimuli. We make no assumptions about particular physiology or cognition, but instead model the process of development/learning from stimuli with feedback from the environment. This is analogous to the experiential learning of any animal whose behaviour is guided visually, and which must learn to resolve perceptual ambiguity in order to survive.

## Results

**Figure 1:**
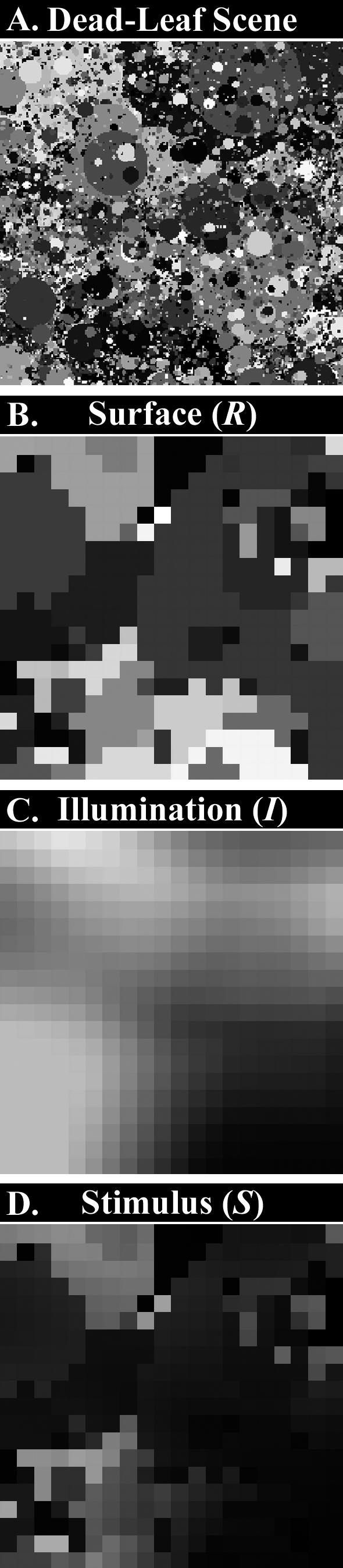
Synthetic “Dead-Leaves” Stimuli (A) “Dead-leaves” example, composed of occluding circular disks with radius r and distribution 1/r^3^. The intensity of each “leaf” is independently drawn from a uniform distribution. (B) Reflectance matrix (*R*), which represents a 20 × 20 subsection randomly chosen from the larger “dead-leaves” stimulus. Typically, between 40 and 60 “leaves” were at least partially visible in each reflectance map. (C) The light falling on a typical surface will come from many sources, so we model illumination with a more gradual change across space than for reflection (see Methods for details). The example illumination matrix (*I*) shown here is a 20 × 20 section chosen from a similar map as *R* but with larger disks than with reflection maps, typically containing 10–15 leaves. These were then heavily blurred producing maps of typically 200–400 distinct levels of intensity, but with a high level of spatial correlation. (D) Stimulus intensity matrix (*S*), which is the pixel-wise product of B and C: *S* = *I* × *R*. All the values are in the range 0…1.

Fifty ANNs were trained using backpropagation to predict the reflectance of surfaces in synthetic scenes, an example of which is shown in Figure 1A. Each scene consisted of a 3-D matrix of 400 matte surfaces (*R*) under spatially heterogeneous patterns of illumination (*I*). As is the case for the human visual system, the trained ANNs did not have direct access to the scenes' reflectance or illumination, but only the product of the two (*R • I* = *S*) at each point in space—thus, the luminance stimulus (*S*) in Figure 1D represents the product of the surface reflectance matrix in Figure 1B and the illumination matrix in Figure 1C. The task was to predict the source reflectance (*R*) of the stimulus (*S*) at the centre of each scene without explicit knowledge of the surface's illumination (*I*).

Surface reflectance matrices (Figure 1B) and illumination matrices (Figure 1C) were created using the “dead-leaves” algorithm, which results in projected images with the same statistical properties as natural images [9]. In 20% of cases, a second surface layer with “gaps” in place of surfaces was placed “in front” of the first surface layer, under independent illumination, equivalent to viewing background objects beyond independently illuminated foreground objects, such as looking through the branches of a tree. See Methods for further details on the ANNs and the “dead-leaves” stimuli.

Note that the ANN training was supervised, meaning that the true target reflectance underlying each stimulus was used by the backpropagation algorithm to estimate errors during learning, which provided feedback for the ANNs. While backpropagation is not physiological in terms of its actual mechanics, the process of altering the network processing according the success and/or failure of its output is equivalent to a visual animal getting feedback from the environment according to the value of its response. Visual systems have evolved to aid survival by allowing animals to respond to the visual environment successfully. This does not necessarily require veridical percepts of the world, but we assume here that generating behaviours that are consistent with surface reflectance, along with other characteristics, will be useful to a visual animal. Animals that generate behaviours that preserve the similarities and differences between surfaces will typically receive some form of feedback from their environment, such as the reward of eating nutritious food or the penalty of eating noxious food. In the same way, “virtual robots” have been shown to develop a form of colour constancy without supervised learning in a visually ambiguous ecology [10]. This feedback can be modelled explicitly using artificial life [10] or reinforcement learning [11], but in this work our focus is not on learning algorithms themselves, but rather on what is encoded. We therefore ignore the temporal credit assignment problem, i.e., the problem of how an animal decides which of its past actions led to a particular reward or penalty. Instead, we consider other sources of uncertainty such as the ambiguity caused by heterogeneous light falling on varied surfaces.

Our emphasis on learning contrasts with “mechanistic” modelling approaches (in the sense defined in [12]), such as the “Anchored Filling-in Lightness Model” [13]. That model describes many of the visual effects discussed here, and more besides, based on neural and anatomical experimental data. It is not derived directly from ecological data in the way that ANNs' behaviour here is, and so cannot give a distal explanation as to *why* such visual behaviours are found. Similarly, the computational Bayesian approach in [12] uses a parametric model, whose form has been chosen manually, whereas the ANNs used here are a nonparametric model, derived entirely from the data.

### Lightness Constancy

After training, each ANN was tested with 10,000 novel images created in the same way as the images in the training set, and the ANN's prediction of the reflectance of each target patch was recorded. The average root-mean-squared (RMS) error for predicted reflectance to the novel test set was 0.171 (with a standard deviation of 0.0016) and the errors approximated a Gaussian distribution (Kolmogorov-Smirnov normality test; *p* ≈ 0). Thus, trained ANNs—like humans—were able to accurately and robustly predict the reflectance of the central surface from uncertain sensory data; i.e., the ANNs exhibited “lightness constancy” (see related work on depth processing [14], the evolution of visually guided behaviour in virtual robots [15], and distance perception [16]; and on perceiving colour constancy by estimating the illumination of a scene using higher-order statistics [17]). Robust response accuracy, however, varied according to the nature of the stimulus. When, for instance, the central target was viewed against a uniform background with uniform illumination (rather than against a fully articulated surround), the RMS error increased significantly to 0.20 (s.d. 0.015; *t*-test: *p* ≈ 0, *n* = 50). An equivalent decrease in lightness (and colour) constancy in low variance scenes is also evident in human perception [18–21]. The study here suggests that this is because increasing the number of surfaces in a scene (i.e., “articulation,” which is a subset of the more general phenomenon of “cue-combination”) narrows the distribution of possible sources of a stimulus, which has been suggested previously in human studies but never tested directly [20,21].

### Brightness Contrast

**Figure 2:**
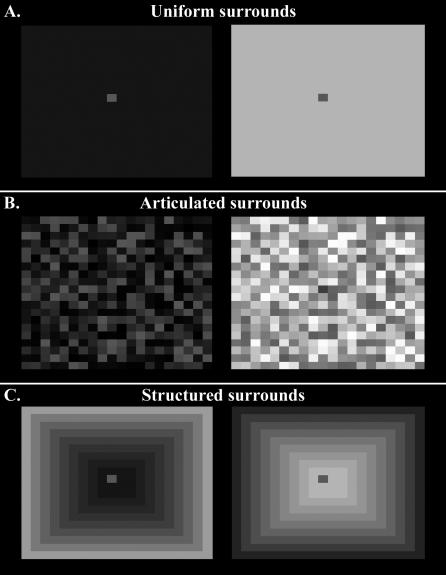
Brightness Contrast Stimuli (A) Simultaneous brightness contrast illusion. See text for explanation. (B) Articulated surrounds with mean *S* = 0.25 and *S* = 0.75 with same target intensity *S* = 0.4. See text for explanation. (C) Concentric rings, both with an average intensity of *S* = 0.5 and a target intensity of *S* = 0.5. In (A–C), ANNs predict a higher reflectance for the stimulus on the left compared to the stimulus on the right.

A basic aspect of human lightness and brightness is that these phenomena do not always accord with stimulus intensity, which is to say we see illusions. The most basic, well-known, and most thoroughly studied illusion is “brightness contrast,” where a central target against a lighter background appears darker than the same target viewed against a darker background (as will be evident to the reader when viewing the two small patches at the middle of the light and dark surrounds in Figure 2A). To test whether trained ANNs also behave in accordance with this illusion, ANNs were presented with “hand-made” stimuli, in which a target stimulus of 0.5 was embedded on uniform surrounds that varied from 0 to 1. The darkest surrounds lead to an average *overestimation* error of 0.36, whereas the lightest surrounds lead to an average *underestimation* error of 0.17. Thus, trained ANNs did indeed exhibit brightness contrast. What is more, the data show that they also exhibited an asymmetry in the relative effects of the darker versus lighter surrounds, with the darker surround “carrying” most of the illusion. Remarkably, this latter asymmetry is also evident in human perception [1,6,22]. The anchoring model [1] explains this in terms of a weighted sum of global and local anchoring and “scale normalisation” effects; however, while that model fits the psychophysical data, it is not predictive as to the strength of the effect, because the weight is never explicitly defined. A probabilistic model more similar to the one here also explains the nonlinear relationship between lightness and intensity in terms of possible real-world sources of an ambiguous stimulus [22], if the relative contributions of reflectance and illumination can be estimated. However, the nonlinearity in brightness contrast, which can be inferred from this model, is symmetrical, not asymmetrical as it is here—and in human perception.

Our model suggests a more explicitly data-driven explanation. We express the reflectance *R* and illumination *I* as fractions of their potential maximum values, so in all cases 0 < *I* < 1 and 0 < *R* < 1. Because the stimulus intensity *S* = *I* • *R*, it is similarly bounded between zero and one. Therefore, the value of *S* defines the minimum possible illumination and reflectance of a target. As an example of this, suppose that *S* = 0.7 in some particular stimulus; the darkest possible value of *R* corresponds to the maximum illumination *I* = 1, giving *R* = 0.7 as the minimum lightness possible. If the exact illumination is unknown, then the bounds are 0.7 ≤ *R* ≤ 1 in this case, and conversely, 0.7 ≤ *I* ≤ 1. In the extreme, if *S* = 1, then *R* = 1 and *I* = 1 are the only possible sources and the stimulus is totally unambiguous. Conversely, images (or parts of images) with low luminance intensity are more ambiguous—i.e., have a wider range of possible scores for *I* and *R*—than high-intensity images. This increased range of possible sources of darker images leads to a greater magnitude of perceptual errors on average, which translates into a larger overestimation of reflectance compared to lighter surrounds on average, assuming that negative values are never predicted. We are not claiming that visual systems must explicitly contain such a model of physics, or that the exact values must be known, but only that past experiences of the consequences of the physics of the environment are encoded in the system, and so behaviour guided by such experiences will lead to the observed patterns of errors.

### Other Lightness Illusions

An important aspect of brightness contrast in humans is that the strength of the illusion is as much a function of stimulus structure as it is of stimulus intensity. For instance, increasing a scene's articulation (as in Figure 2B) increases human perception of brightness contrast considerably [23,24]. Similarly, when presented with targets on two fully articulated surrounds (one light, one dark), the difference in the predicted reflectance of the identical targets was increased (Figure 2B). Also, altering the spatial configuration of a target's surround, without altering the average luminance, can create the illusion of brightness contrast. When the nets were presented with targets on surrounds of identical average intensity, but of differing spatial structure (Figure 2C), they continued to *underestimate* the target on a local light surround, and *overestimate* the target in a local dark surround much like humans, specifically outputting *R* = 0.32 (0.025) and *R* = 0.74 (0.011), respectively, for the images shown in Figure 2C. The papers summarised in [23] discuss various aspects of articulation in detail, including the effect of both the number of surfaces in a scene and their structural organisation. Similarly here, it is not simply increasing the number of surfaces that leads to better constancy (and so to smaller errors), but the structure of the articulation. More specifically, what matters is past experience regarding the probable source of that articulated information, as has been suggested previously [23].

**Figure 3:**
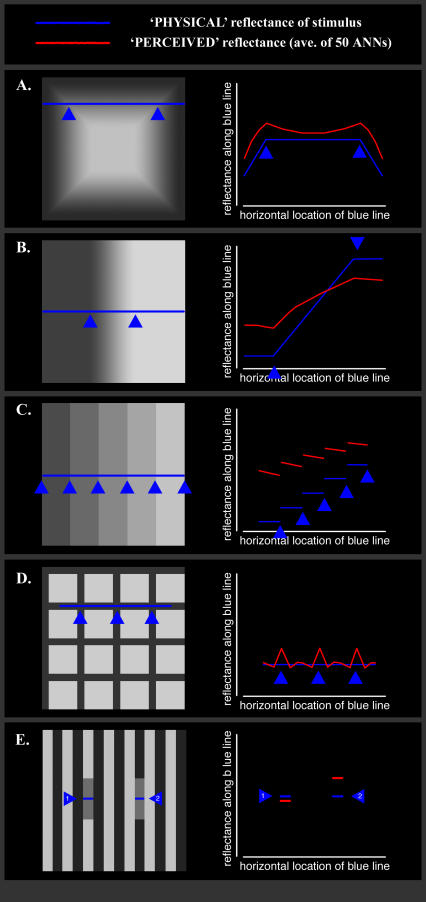
Artificial Neural Network Responses to Optical Illusions (A) Vasarely illusion stimulus on left. Note the illusory regions of lightness along the diagonals formed by the corners of the concentric squares. Blue line on right indicates the intensity profile at the corresponding points of the blue line on the left. Red line shows the relative reflectance predicted by the networks. Units are arbitrary and so are not plotted throughout this figure. (B) Mach band stimulus consisting of a dark bar (left) and a light bar (right) with a linear gradient between. Blue line on right indicates the intensity profile at the corresponding points of the blue line on the left. Red line shows the relative reflectance predicted by the networks. (C) Chevreul illusion stimulus, with five bars of uniform reflectance. Blue line on right indicates the intensity profile at the corresponding points of the blue line on the left. Red line shows the relative reflectance predicted by the networks. (D) Hermann grid illusion stimulus. Blue line on right indicates the intensity profile at the corresponding points of the blue line on the left. Red line shows the relative reflectance predicted by the networks. (E) White's illusion on left. The grey areas indicated have the same physical reflectance, although the left-hand one appears darker than the other. The ANN response on the right corresponds to this human experience, with the first perceived reflectance (1) being darker than the second (2).

The ANNs were next tested on other, more complex but well-known brightness contrast–like phenomena, specifically the Vasarely illusion, Mach bands, Chevreul patterns, and the Hermann grid. In the Vasarely illusion (Figure 3A), the corners of each repeated square appear brighter than their immediate surround (which results in what looks like a four-edged star), even though the stimulus is uniform at these junctions. In Mach bands (Figure 3B), a linear gradient appears to be flanked by a highlight at the lightest end of the gradient and a “lowlight” at the gradient's minimum. Neither of these features actually exists in the intensity profile of the stimulus. In Chevreul patterns (Figure 3C), uniform bars appear graded in lightness. And in the Hermann grid (Figure 3D), light spots appear at the central junction of the dark lines where no light dot actually exists. The 50 trained ANNs were presented with each of these stimuli in turn, none of which were presented during training. Their average response is shown in the corresponding row of the right column in Figure 3A–3D. By comparing the stimulus' intensity profile (red line) with the nets' response profile (blue line) at each corresponding point, it is clear that, as before, the networks exhibit responses that are qualitatively similar to human perception in each instance. (Whether they are quantitatively similar to human perception is not relevant, given the inevitable differences in complexity between natural ecology and the “dead-leaves” ecology.)

### White's Illusion

The results thus far are consistent with the hypothesis that human illusions of lightness are caused by nothing more (or less) than image ambiguity and its empirical—and thus statistical—resolution. The above contrast illusions, however, are also consonant with many other models predicted on, for instance, the statistics of natural images or assumption about low-level and mid-level processing [1,5,24–25]. Indeed, any model that incorporates lateral inhibitory connections, such as centre/surround receptive fields, will predict most of the above phenomena (e.g., [5,25]), which is the typical explanation in most neuroscience textbooks. Few explanations, however, can simultaneously predict both brightness contrast (including its asymmetry) and brightness assimilation—e.g., White's illusion—without recourse to one or more adjustable free parameters [25]. (Important exceptions include the filter model discussed previously [5] and a statistical approach which uses a database of natural scenes to estimate probability distributions over structures in lightness stimuli, including White's stimulus [26].) What makes these two illusions difficult to reconcile simultaneously is that they are diametrically opposed to one another. In brightness contrast, the target on a dark surround appears lighter than the same target on a light surround (Figure 2A), whereas the opposite is true for assimilation in general and White's illusion in particular: the target on the overall darker local surround appears *darker* (not lighter) than the same target on the overall lighter local surround (Figure 3E; see [1] for an elegant description of these phenomena and their current explanations). White's stimulus can be interpreted as a series of vertical dark and light bars partially obscuring a pair of mid-grey bars on a monochrome background.

Here, the trained ANNs exhibited both brightness contrast ***and*** White's illusion (see right column of Figure 3E). As always, the emergent behaviour of the ANNs can be explained in terms of the statistics of their visual experience. Of particular relevance is their experience with the 3-D layering of the surfaces in space. A separate group of ANNs was trained using scenes composed of surfaces in only one depth plane, consisting of the same “dead-leaves” images described in the Methods section, but without the separate mask layer on any of the stimuli. Compared to the main group of ANNs, these lost the “ability” to see White's illusion, but maintained the ability to see lightness constancy, brightness contrast, and related phenomena (unpublished data). Thus, when presented with surfaces at different depth planes under independent illumination, the ANNs learned to ignore information arising from surfaces that were not co-planar with the target; since illumination of each depth-plane is independent, only co-planar information provides statistical information about the probable source of the target. Thus, changing the ecology (by introducing layers using masks) leads directly to a change in behaviour (the ANNs' response to White's stimulus) showing a causal link between the two.

**Figure 4:**
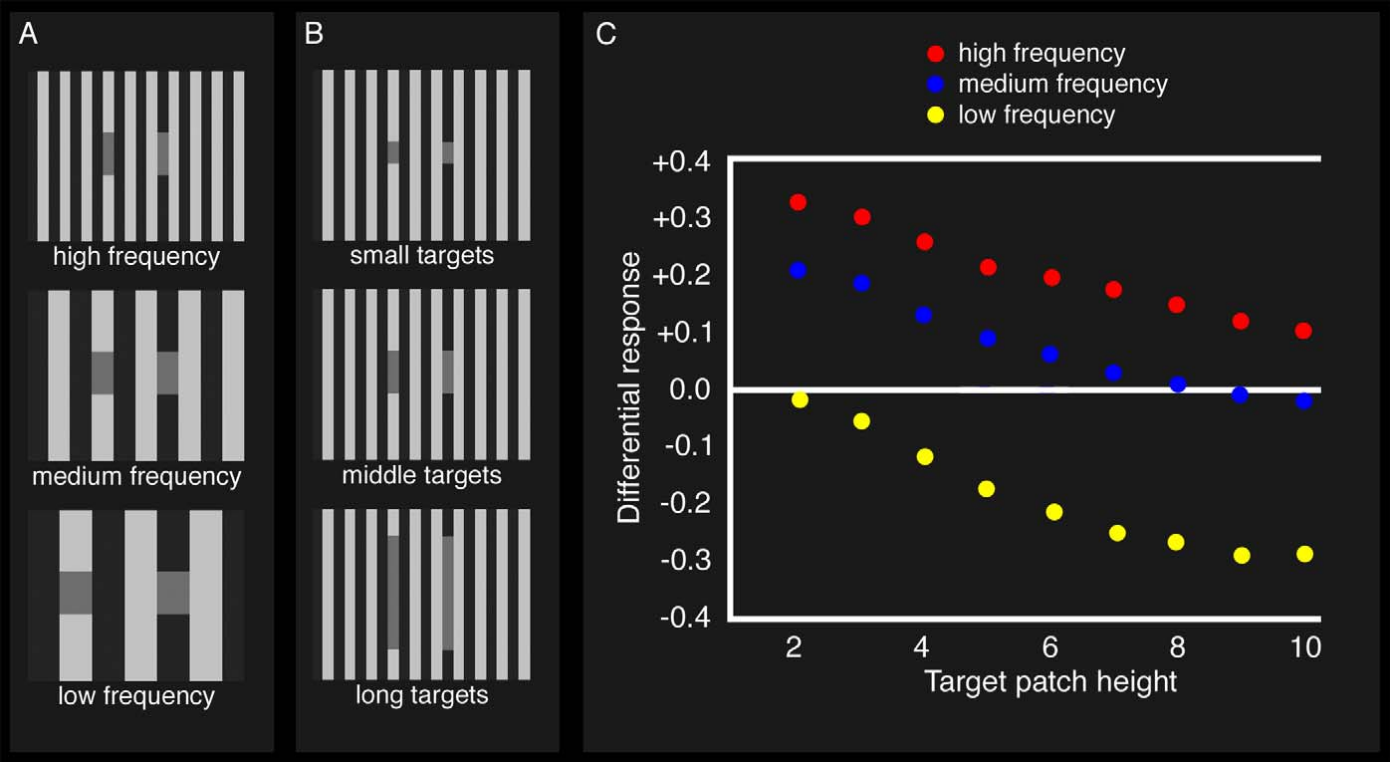
White's Stimuli and ANN Responses (A) Three White's stimuli of varying spatial frequency and (B) three White's stimuli with different target patch heights. In all cases, the left-hand target patch has the same intensity as the right-hand patch, but generally appears darker to humans. The stimuli seen by the ANNs are 20 × 20 pixels. (C) Mean ANN responses to White's stimuli of varying frequencies with varying test patch heights. Each value is the difference in predicted reflectance for the two test patches. A positive difference means that the test patch on the light bar appears darker than the test patch on the dark bar; a negative difference means the test patch on the light bar appears *lighter* than the patch on the dark bar. The former is consistent with White's illusion, the latter with brightness contrast. The results show i) that decreasing the frequency of the background stripes (i.e., making them wider) also decreases the strength of White's illusion; and also ii) that increasing the height of the test patch decreases the strength of White's illusion. Both results correspond to human psychophysical responses [5].

It is important to emphasise, however, that while White's illusion only arises when the networks had experience of 3-D scenes, this is not equivalent to saying that the networks “represented” depth in their post-receptor processing. Indeed, it is highly unlikely that the networks encode depth information explicitly, or indeed contour junction cues, as has been posited for human visual processing, since varying the spatial frequency of the stimulus or the height of the individual test patch varies the strength of the illusory response (see Figure 4A and 4B, respectively) without altering the stimulus' junctions. More specifically, decreasing the spatial frequency of the stimulus and/or target height decreases the ANNs' perception of White's illusion without altering the stimulus' junctions. Remarkably, these latter two observations have also been made of human perception of White's stimulus [5].

### Seeing without Context

Not all human lightness illusions are a consequence of spatial context, and in these cases we found further similarities between the ANN's behaviour and human visual perception. For instance, when viewed in a “void” (i.e., on a black surround), the relationship between a surface's stimulus and its (human-) perceived lightness is not linear, but follows the power law ψ(*S*) = *kS* ^α^, where ψ(*S*) is the perceived lightness, *S* is the physical intensity of the stimulus, *k* is a scaling constant, and α is the exponent that describes the shape of the relationship to perceived lightness. For humans, the value of the exponent α typically varies between 0.33 to 0.5 in different studies [27]. When the ANNs are presented with uniform images of increasing intensity, the relationship between target intensity and predicted reflectance also follows a power law with an exponent (α) that equals 0.334—broadly similar to humans.

## Discussion

The ANNs used here are structurally unlike the human visual system: they are smaller and less complex; they lack recurrent connections, spiking, adaptation (after learning is complete), and so on; they are nonhierarchical, and so cannot generate behaviours according to so-called “top-down,” “mid-level,” or “cognitive” influences on “bottom-up” processing. Indeed, these ANNs lack all the proximal mechanisms that are usually thought to be the immediate cause of human visual illusions. Instead, the output of each ANN is driven solely by the statistics of its training history instantiated in the functional architecture of its network. Though sometimes seen as a drawback, this simplicity is taken advantage of to rationalise human illusions, not by modelling what is currently known of human perception and/or primate neurophysiology, but by modelling the inherent ambiguity of human and nonhuman visual ecology that all natural systems must solve to survive, and its empirical resolution. This extends several recent studies that have found relationships between the statistics of images of natural scenes and human perception (e.g., [25–26,28–30]). We can begin to move from correlative to causative explanations.

Perception can be defined as the process of acquiring and organising information from sensors. The input nodes of the ANNs are presented with images in terms of the luminance intensity across space, from which the ANNs must extract scene information, specifically the reflectance of a target patch. This is equivalent to one of the many tasks that the human visual system performs. The *Oxford English Dictionary* defines an illusion as “something that deceives or deludes by producing a false impression.” Every instrument has measurement errors and the human visual system is no exception. So *every* percept will have an error associated with it, be it large or small. Errors in visual perception are defined as the difference between what is seen and what the actual physical quality of the retinal stimulus with which the percept is associated is [1], irrespective of whether the physical source is ever known. Given this definition, a so-called “illusory image,” such as the stimuli in Figure 3, is one that induces perceptions that deviate from the underlying reality of the image, a view consistent with recent Bayesian frameworks of constancy (e.g., [12]). There is, however, no absolute threshold on these errors that defines a percept as illusory or non-illusory. We must therefore consider the magnitude of perceptual errors and relate these to the past experiences of the observer.

**Figure 5:**
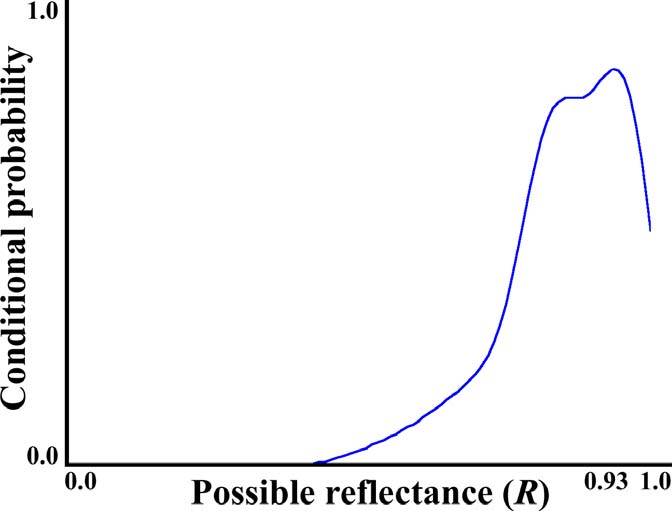
Conditional Probability Distribution of Reflectance Given Past Experience and a Particular Stimulus as Context A maximum-likelihood estimation allows the observer to predict the target reflectance and will be correct (approximately) most of the time. If the true reflectance actually lies in a low-likelihood tail of the distribution, then the resulting percept is an illusion.

Returning to the ANNs used earlier, recall that when shown novel “dead-leaves” images, the RMS error was 0.171. Furthermore, approximately 79% of the predictions were within ±0.2 of the target, and just 1% of the errors were greater than ±0.5; i.e., most images were interpreted approximately correctly, but none perfectly. The equivalent error for simultaneous brightness contrast (Figure 2A), with a mid-grey patch on a black background, was 0.36 (s.d. 0.032), an unusually large error. As a specific example, Figure 5 shows the range of all possible reflectances of a single target patch (*x*-axis) and their relative probabilities (*y*-axis), for a single “dead-leaves” stimulus. The probabilities are derived from the past experiences of a single ANN, and the peak on the curve corresponds to a reflectance (*R*) of 0.93. This is by definition the “most likely source” of this particular stimulus in the ANN's past visual experience. If the *actual* reflectance of the stimulus under consideration is close to the most likely source of the stimulus (i.e., a surface with a reflectance close to 0.93), then the prediction/percept is “correct” and we would say that lightness constancy holds. One would also say that the percept is not an illusion. If, on the other hand, the actual reflectance happened not to be near the most likely source of the stimulus (i.e., more than or less than 0.93), then while the predicted reflectance would have been “correct” most of the time, it would be “wrong” *in this particular instance*, lightness constancy would have failed, and the percept would be called an illusion. What is more, the further into the tail of “unlikeliness” the source of the stimulus is, the more “illusory” the percept becomes, suggesting that illusions of lightness and lightness constancy exist on a continuum, as opposed to being fundamentally different kinds of phenomena.

It is therefore misleading to describe any stimulus as being an illusion in isolation. Instead, one can describe the true source of a stimulus as being unlikely given the past experiences of a particular observer, and therefore likely to induce an erroneous percept in that observer. Given the similarity of the shared experiences of humans, and our shared genes, it should not be surprising that the patterns of errors that we make are also shared. The exact distribution of errors for human or animal perception is hard to quantify, and the factors leading to more or less lightness constancy are largely unknown [23]. However, it seems clear that most responses are approximately correct, at least where it is possible to measure the true source, although constancy does fail significantly in some cases. The nearest human psychophysical study that we are aware of measures colour constancy for coloured papers under varying illumination [31]. They define a constancy index that ranges from 0 for no constancy to 1 for perfect constancy, the latter meaning that a surface colour is perceived according to the surface spectral properties alone (and not illumination, for example). They measure a colour constancy index of around 0.8, although in many experiments the index was much lower. Constancy can be seen as the inverse of illusions, if we assume that the constant response has a small error under a range of illuminations, and illusions generate large errors. The errors of the ANNs suggest a similar magnitude of constancy, although direct comparison between such different measures is never ideal. We know of no such score for lightness constancy under typical, natural conditions, but it is reasonable to suppose a broadly similar continuous distribution exists for humans, too.

In conclusion, the emergent similarity between human perception and the ANNs' output provides direct support for the view that illusions are caused by (as opposed to merely correlated with) the statistics of past visual experience towards surfaces in space under spatially heterogeneous illumination given ambiguous image data. Because stimulus ambiguity is an inherent challenge of natural visual ecology, illusions must also be inevitable in nature, suggesting that human illusions are common to all visual animals despite vast differences in their underlying neural machinery, which has important consequences for thinking about the biological and computational principles of vision. Evolving or training synthetic systems in ecologically relevant environments provides an important new strategy for uncovering what these principles are that usefully map images to scenes according to the statistics of experience. Finally, the study provides a clear description of what an illusion is, and why we see them: an illusion describes the condition in which the actual source of a stimulus differs from the stimulus' most likely source given the observer's past experience.

## Methods

### Artificial neural networks.

The ANNs used here were standard multilayer perceptrons trained via backpropagation. We use multilayer perceptrons because they are known to be universal approximators, capable of learning arbitrary mappings from a finite set of examples. In preliminary experiments, we achieved similar results using support vector regression methods (unpublished data), and believe that any suitable powerful nonlinear multivariate regression tool would work as well. The behaviour we describe is ultimately due to the data, not the learning algorithm.

Each ANN had 400 inputs nodes, one for each pixel of the stimuli; four hidden nodes in one layer; and one output node. The training was supervised, so the target reflectance in the training images was used to estimate errors during the training. The output was therefore the ANN's prediction of the reflectance of the central target patch of the stimulus presented to it. The inputs consisted solely of the stimulus intensity, and not reflection or illumination explicitly. All nodes were fully connected to nodes of their adjacent layers; there were no connections between nodes of the same layer; and connection weights could be positive or negative. Each ANN was initialised with random weights, then trained for 150 iterations with 20,000 training images. These parameters were chosen based on preliminary experiments, and are not critical. Many factors are known to affect the performance of ANNs, such as the number of hidden nodes, the learning rate, the number of training iterations (see Figures S1 and S2), the number of training examples, and so on. Furthermore, these factors tend to interact, making any exhaustive analysis effectively impossible, and making it difficult to guarantee that any particular ANN is “optimal.” However, our aim here is not optimality, but is rather to show that the results described in the paper are robust, and, to demonstrate this, we now briefly analyse some of these parameter settings.

All nonparametric learning systems, including ANNs trained by backpropagation, are prone to “overfitting,” when they accurately model the data that they are trained with, but fail to generalise well to novel data. One conventional solution is to stop training after a fixed number of iterations, before this problem arises, which is why we limit the training algorithm to 150 iterations (see Figure S1).

To see the effect of varying the number of hidden nodes, we trained a series of ANNs, each containing between one and 50 nodes in a single hidden layer. The minimum error corresponds to ANNs with four nodes in their hidden layer (see Table S1). However, a series of *t*-tests indicate that the other ANNs achieved performances that were not significantly different (*p* > 0.05 in all cases). Thus, the choice for the number of nodes is somewhat arbitrary, reinforcing the notion that it is the statistics of the training set that are critical, rather than the fine details of the learning algorithm.

Next we considered the number of training records used by the backpropagation algorithm. Again, we trained a series of ANNs with sets of novel “dead-leaves” stimuli. Each ANN had four hidden nodes, but the number of training records varied from 333 to 20,000. As expected, being given more training examples allowed the ANNs to achieve a lower test error, because each independent training example provides extra information about the underlying function (see Table S2). Given the trend of decreasing returns, increasing the number of records above 20,000 would make only a marginal difference, with the cost of longer training times.

Each node of an ANN contains an activation (or “transfer”) function, which takes the sum of the inputs and transforms it, typically rescaling the value to a fixed range. A typical activation function, which we use in the ANNs described in the main paper, is the log sigmoid function, which produces values in the range [0, 1]. The tan sigmoid function, which produces values in the range [−1, +1] and the linear transfer function, which produced unbounded values, were also used in new ANNs for comparison. As the errors in Table S3 show, there is no significant difference between log sigmoid and tan sigmoid functions, as expected. The pure linear activation function, which gives no bounds on the outputs, leads to significantly worse performance. Thus the choice of a particular activation function is not critical, although in the extreme case of a linear function, learning is considerably degraded.

We also tested some of these alternative ANNs with the various “illusion” stimuli used elsewhere in the paper. As a simplified measure of different ANNs responses to the test “illusory” stimuli, we measured each ANN's predicted reflectance for the test patches in the brightness contrast, Hermann grid, and White's stimuli (see Figures 2A, 3D, and 3E, respectively). For each stimulus, we selected two pixels that had identical reflectance values but generate illusory responses in humans. For each pair, we calculated the difference in the ANN's response, such that a score of zero means that they do NOT perceive any illusion, and a positive score corresponds to human perceptions. (This is the same differential measure used in Figure 4C.) The larger the positive score, the stronger the illusion is perceived. Negative scores indicate the “opposite” of human perception. While there is no direct relationship between the magnitudes and human perception, they do provide an indication of the strengths of the illusions for the ANNs. The overall effect is that as training proceeds, the error drops and the strength of the illusions increases (Figure S2). This again shows that the appearance of illusions is causally related to solving the lightness constancy problem.

All experiments were carried out on a standard desktop PC using Matlab 6.5 (Mathworks) and the Matlab Neural Networks toolbox version 4.

### “Dead-leaves” images.

A number of 200 × 200 pixel “dead-leaves” images were created following the algorithm presented by Lee et al. [9], which produces images with similar statistics as those that have been found in a wide range of natural scenes. The implementation we used was based on Matlab code provided in the Toolbox Signal (2006) by Gabriel Peyré. Each image was composed of a large number of partially occluding achromatic disks, which can be thought of as a series of “dead leaves” falling on top of each other. The leaf radius is distributed as 1/r^3^, so these images tend to have a few large “leaves” and many smaller ones, much as with natural scenes. For presentations to the ANNs, random 20 × 20 pixel samples were selected from these large images. The minimum-sized disk was fixed at 0.002 for the reflection maps and 0.01 for the illumination maps. The latter were blurred by filtering with a Gaussian filter of size 8 × 8 with a width of 15. The stimulus matrix presented to the ANNs is defined as *S* = *I* • *R*. Both *I* and *R* (and therefore *S*) are scaled in the range 0…1. Where a second layer was used to create 3-D stimuli (in one-fifth of the training set), the same procedure was used to create the surfaces and the illumination. The layer was then reduced to a series of random horizontal and vertical strips covering an average of 10% of the image opaquely. The remaining 90% was unchanged. The target could be in either layer. We have not carried out any human psychophysical experiments testing responses to these stimuli; however, the algorithm is designed to generate images that are statistically similar to natural scenes, so we assume that human responses would be quite consistent with responses to natural scenes.

Preliminary work showed that if the distribution of the reflectance and illumination maps were very similar, then the ability to resolve lightness constancy in the ANNs was reduced, though not abolished (unpublished data). Presumably, this is because every stimulus was so ambiguous that resolution was increasingly difficult. Given that humans and other animals can solve lightness constancy at least most of the time, the real visual ecology must provide enough information to allow the disambiguation to take place. In our simplified model, this is achieved by ensuring that the distributions of *R* and *I* are sufficiently different.

These “dead-leave” images, with heterogeneous light and partial masking, represent a simple model ecology. The size of the distinct surfaces within each scene follows the same distribution as found in natural scenes. The illumination is assumed to come from multiple sources, consistent with some light being reflected from nearby surfaces. The reflectance map is therefore approximately piecewise constant, while the illumination map only changes smoothly, as in [4] and elsewhere. The addition of a second “masking” layer aims to simulate effects such as the viewer looking through the branches of a tree or through a windowframe. Such a simple model could be extended in many ways to make it more natural and realistic, such as added colour, transmittance effects, depth, objects of varied shape with or without attached shadows, sharp shadow edges, and so on. Several of these are the subject of ongoing work, which will allow a wider range of visual behaviours to be studied, such as testing the models' response to colour illusion stimuli. Similarly, we have chosen not to model the eye explicitly, such as defining cone response functions or light adaptation, instead concentrating on the more generic aspects of learning to respond to a visual ecology.

## Supporting Information

Figure S1 Overfitting Caused by Training the ANN for Too Many Epochs The error on the training set continues to drop as the backpropagation algorithm continues, but the test error on novel “dead-leaves” images starts to rise after around 150–200 epochs. This overfitting is a problem with any nonparametric learning algorithm, such as ANNs, and a typical solution that we adopt is to stop training after a fixed number of iterations (150).
(36 KB DOC)

Figure S2 ANNs' Response to Various Stimuli during Training For each test stimulus, we selected two pixels that had identical reflectance values but generate illusory responses in humans. For the brightness contrast and White's stimuli, we used the pair of test mid-grey patches, and for the Hermann Grid we used an “intersection” pixel and an “edge” pixel halfway between two intersections. The RMS error is the usual test against a novel set of “dead-leaves” images. As training continues, the test error drops (left axis) and the strength of the illusory percepts tends to increase (right axis).
(383 KB DOC)

Table S1 The Effect of the Number of Hidden Nodes None of the test errors is significantly worse than the optimum, corresponding to four hidden nodes (two-tailed *t*-test; *p* > 0.05 in all cases). In each case, the number of training epochs was adjusted to minimise the test error.
(31 KB DOC)

Table S2 The Effect of the Number of Training Records on ANNs with Four Hidden Nodes Providing more training examples leads to lower test errors, at a decreasing rate.
(29 KB DOC)

Table S3 Various ANN Activation Functions Two-tailed *t*-tests show that: log sigmoid is *not* significantly different to tan sigmoid (*p* = 0.067); log sigmoid is significantly better than pure linear (*p* ≈ 0); tan sigmoid is significantly better than pure linear (*p* ≈ 0).
(26 KB DOC)

## Abbreviations

ANN: artificial neural network
RMS: root-mean-squared
