# Supplementary material for: What Are Lightness Illusions and Why Do We See Them?
Source: PLoS Comput Biol. 2007 Sep 28;3(9):e180. doi: 10.1371/journal.pcbi.0030180 (PMC1994982; doi:10.1371/journal.pcbi.0030180)
Supplement: Figure S2 — For each test stimulus, we selected two pixels that had identical reflectance values but generate illusory responses in humans. For the brightness contrast and White's stimuli, we used the pair of test mid-grey patches, and for the Hermann Grid we used an “intersection” pixel and an “edge” pixel halfway between two intersections. The RMS error is the usual test against a novel set of “dead-leaves” images. As training continues, the test error drops (left axis) and the strength of the illusory percepts tends to increase (right axis). (383 KB DOC) [file pcbi.0030180.sg002.doc]

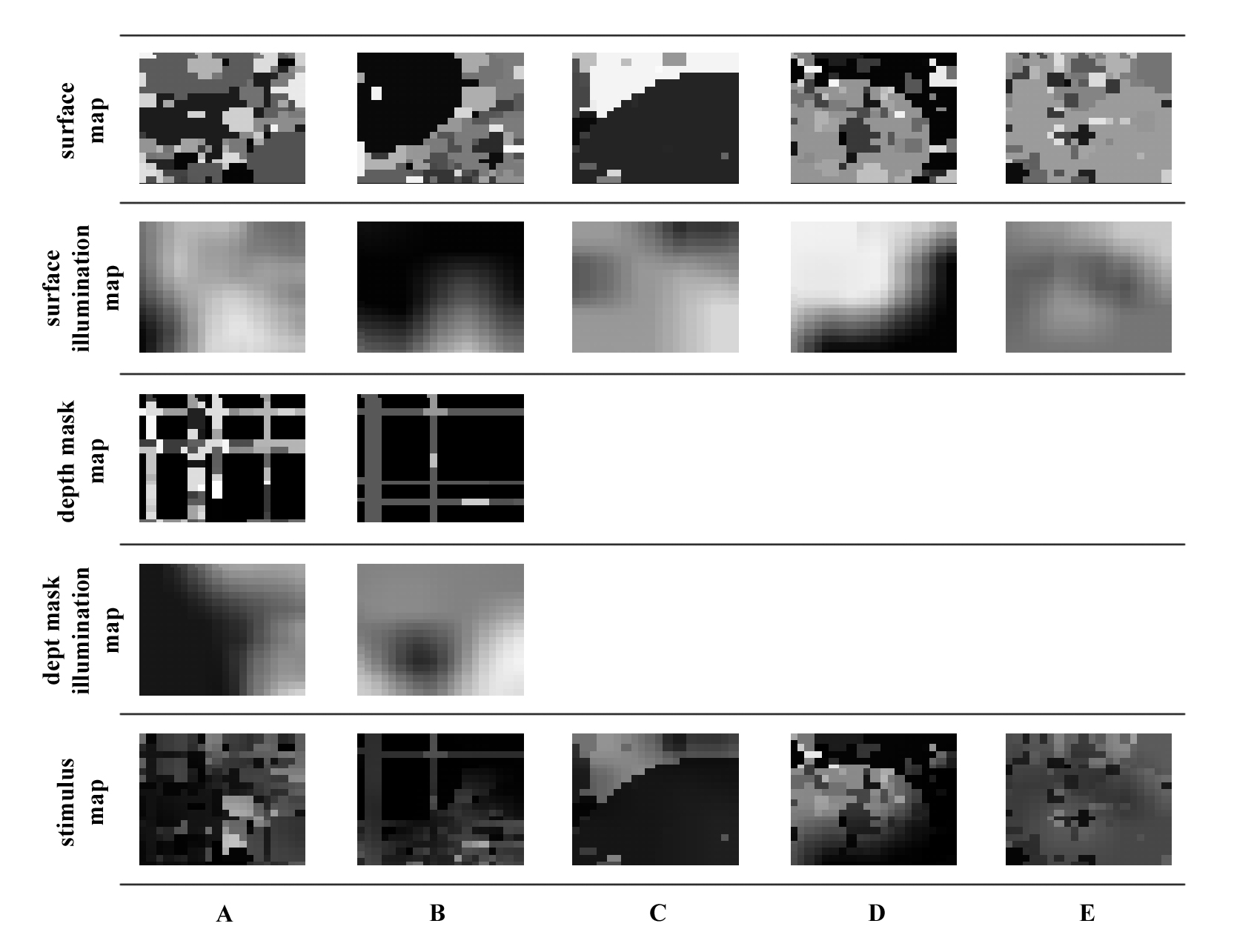


Figure S3: Example dead leaves stimuli and sources. Each column shows a single stimulus below its source components. From top to bottom, row 1 is the reflectance map; row 2 is the illumination map; row 3 is the mask reflectance (if present); row 4 is the mask illumination (if present); row 5 is the stimulus map.

In the full training sets, 20% of images have a mask present, as in stimuli a) and b) here. The product of the “mask reflectance” and the “mask illumination” is superimposed onto the background stimulus, itself being the product of the background reflectance and background illumination, to produce the final stimulus intensity map (bottom row).
