# Supplementary material for: What Are Lightness Illusions and Why Do We See Them?
Source: PLoS Comput Biol. 2007 Sep 28;3(9):e180. doi: 10.1371/journal.pcbi.0030180 (PMC1994982; doi:10.1371/journal.pcbi.0030180)
Supplement: Table S1 — None of the test errors is significantly worse than the optimum, corresponding to four hidden nodes (two-tailed t-test; p > 0.05 in all cases). In each case, the number of training epochs was adjusted to minimise the test error. (31 KB DOC) [file pcbi.0030180.st001.doc]

| *Number of hidden nodes* | *Test RMS error* |
| --- | --- |
| 1 | 0.1712 |
| 2 | 0.1715 |
| 3 | 0.1711 |
| 4 | 0.1700 |
| 5 | 0.1713 |
| 10 | 0.1709 |
| 20 | 0.1717 |
| 50 | 0.1714 |
