# Supplementary material for: What Are Lightness Illusions and Why Do We See Them?
Source: PLoS Comput Biol. 2007 Sep 28;3(9):e180. doi: 10.1371/journal.pcbi.0030180 (PMC1994982; doi:10.1371/journal.pcbi.0030180)
Supplement: Table S2 — Providing more training examples leads to lower test errors, at a decreasing rate. (29 KB DOC) [file pcbi.0030180.st002.doc]

| *Training records* | *RMS Error* |
| --- | --- |
| 333 | 0.248 |
| 667 | 0.230 |
| 3333 | 0.183 |
| 6667 | 0.177 |
| 13333 | 0.174 |
| 20000 | 0.171 |
