# Supplementary material for: What Are Lightness Illusions and Why Do We See Them?
Source: PLoS Comput Biol. 2007 Sep 28;3(9):e180. doi: 10.1371/journal.pcbi.0030180 (PMC1994982; doi:10.1371/journal.pcbi.0030180)
Supplement: Table S3 — Two-tailed t-tests show that: log sigmoid is not significantly different to tan sigmoid (p = 0.067); log sigmoid is significantly better than pure linear (p ≈ 0); tan sigmoid is significantly better than pure linear (p ≈ 0). (26 KB DOC) [file pcbi.0030180.st003.doc]

| *Function* | *RMS Error* |
| --- | --- |
| Log sigmoid: | 0.1699 |
| Tan sigmoid: | 0.1711 |
| Pure linear: | 0.2306 |
