# Supplementary material for: What Are Lightness Illusions and Why Do We See Them?
Source: PLoS Comput Biol. 2007 Sep 28;3(9):e180. doi: 10.1371/journal.pcbi.0030180 (PMC1994982; doi:10.1371/journal.pcbi.0030180)
Supplement: Supplementary file 5 [file pcbi.0030180.sg001.doc]

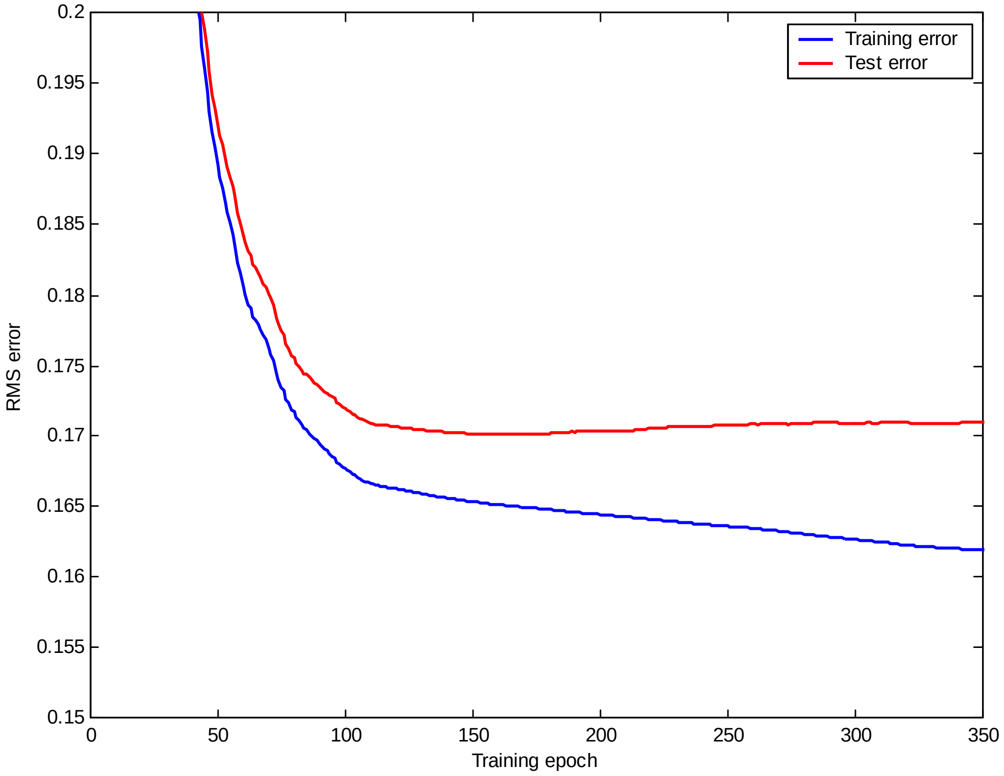
Training error
Test error
RMS error
Training epoch
0.2
0.195
0.19
0.185
0.18
0.175
0.17
0.165
0.16
0.155
0.15
0
50
100
150
200
250
300
350
